# Supplementary material for: A cross-country comparison of malaria policy as a premise for contextualized appropriation of foreign aid in global health
Source: Health Res Policy Syst. 2021 Jun 14;19:93. doi: 10.1186/s12961-021-00700-6 (PMC8201720; doi:10.1186/s12961-021-00700-6)
Supplement: Supplementary file 1 — Additional file 1. Small countries and dependent territories. [file 12961_2021_700_MOESM1_ESM.doc]

Additional file 1: Small countries and dependent territories

| American Samoa | Gambia | Qatar |
| --- | --- | --- |
| Andorra | Grenada | Reunion |
| Anguilla | Guadeloupe | Saint Helena |
| Antigua and Barbuda | Guam | Saint Kitts and Nevis |
| Aruba | Guinea-Bissau | Saint Lucia |
| Bahamas | Isle of Man | Saint Pierre and Miquelon |
| Bahrain | Kiribati | Saint Vincent and the Grenadines |
| Barbados | Liechtenstein | Samoa |
| Belize | Luxembourg | San Marino |
| Bermuda | Macau | Sao Tome and Principe |
| British Virgin Islands | Maldives | Seychelles |
| Brunei | Malta | Solomon Islands |
| Cape Verde | Marshall Islands | Swaziland |
| Cayman Islands | Martinique | Timor |
| Comoros | Mauritius | Tokelau |
| Cook Islands | Micronesia | Tonga |
| Cyprus | Monaco | Trinidad and Tobago |
| Djibouti | Montserrat | Turks and Caicos Islands |
| Dominica | Nauru | Tuvalu |
| Equatorial Guinea | Netherlands Antilles | United States Virgin Islands |
| Falkland Islands | New Caledonia | Vanuatu |
| Faroe Islands | Niue | Wallis et Futuna |
| Fiji | Northern Mariana Islands |  |
| French Polynesia | Palau |  |
