# Supplementary material for: A cross-country comparison of malaria policy as a premise for contextualized appropriation of foreign aid in global health
Source: Health Res Policy Syst. 2021 Jun 14;19:93. doi: 10.1186/s12961-021-00700-6 (PMC8201720; doi:10.1186/s12961-021-00700-6)
Supplement: Supplementary file 2 — Additional file 2. Data sources. [file 12961_2021_700_MOESM2_ESM.doc]

# Additional files

Additional file 1: Small countries and dependent territories

| American Samoa | Gambia | Qatar |
| --- | --- | --- |
| Andorra | Grenada | Reunion |
| Anguilla | Guadeloupe | Saint Helena |
| Antigua and Barbuda | Guam | Saint Kitts and Nevis |
| Aruba | Guinea-Bissau | Saint Lucia |
| Bahamas | Isle of Man | Saint Pierre and Miquelon |
| Bahrain | Kiribati | Saint Vincent and the Grenadines |
| Barbados | Liechtenstein | Samoa |
| Belize | Luxembourg | San Marino |
| Bermuda | Macau | Sao Tome and Principe |
| British Virgin Islands | Maldives | Seychelles |
| Brunei | Malta | Solomon Islands |
| Cape Verde | Marshall Islands | Swaziland |
| Cayman Islands | Martinique | Timor |
| Comoros | Mauritius | Tokelau |
| Cook Islands | Micronesia | Tonga |
| Cyprus | Monaco | Trinidad and Tobago |
| Djibouti | Montserrat | Turks and Caicos Islands |
| Dominica | Nauru | Tuvalu |
| Equatorial Guinea | Netherlands Antilles | United States Virgin Islands |
| Falkland Islands | New Caledonia | Vanuatu |
| Faroe Islands | Niue | Wallis et Futuna |
| Fiji | Northern Mariana Islands |  |
| French Polynesia | Palau |  |

Additional file 2: Data sources

| Variable * | Source | Reference |
| --- | --- | --- |
| population within 100 kilometres of coast, latitude of country centroid, elevation, landlocked country, openness, international country risk guide index | Center for International Development | www.cid.harvard.edu |
| land area, population, population density, urban population, total GDP, GDP per capita, government health to total government expenditure, official development assistance received per capita | World Bank | data.worldbank.org/indicator |
| total health expenditure per capita, malaria incidence per 1000 population at risk, funding for malaria control, ITNs/LLINs are distributed free of charge, ITNs/LLINs are distributed to all age groups, ITNs/LLINs distributed through mass campaigns to all age groups, IRS is recommended by malaria control programme, DDT is used for IRS, IPTp is used to prevent malaria during pregnancy, seasonal malaria chemoprevention (SMC or IPTc) is used, patients of all ages should get diagnostic test, malaria diagnosis is free of charge in the public sector, RDTs are used at community level, G6PD test is recommended before treatment with primaquine, ACT for treatment of *Plasmodium falciparum*, pre-referral treatment with quinine or artemether IM or artesunate suppositories, single dose of primaquine is used as gametocidal medicine for *Plasmodium falciparum*, Primaquine is used for radical treatment of *Plasmodium vivax* cases, directly observed treatment with primaquine is undertaken | World Health Organization | www.who.int /malaria/publications |
| HDI | United Nations Development Programme | hdr.undp.org/en/data |
| island | Integrated Island Database | www.island-database.uni-hamburg.de |

* ACT: artemisinin-based combination therapy; DDT: dichloro-diphenyl-trichloroethane; G6PD: glucose-6-phosphate dehydrogenase; GDP: gross domestic product; HDI: human development index; IM: intramuscular; IPTc: intermittent preventive treatment in children; IPTp: intermittent preventive treatment in pregnancy; IRS: indoor residual spraying; ITN: insecticide-treated mosquito net; LLIN: long-lasting insecticidal net; NMCP: national malaria control programme; RDT: rapid diagnostic test; SMC: seasonal malaria chemoprevention.

Additional file 3: Correlation matrix of policy scores, malaria, social, economic and environmental parameters


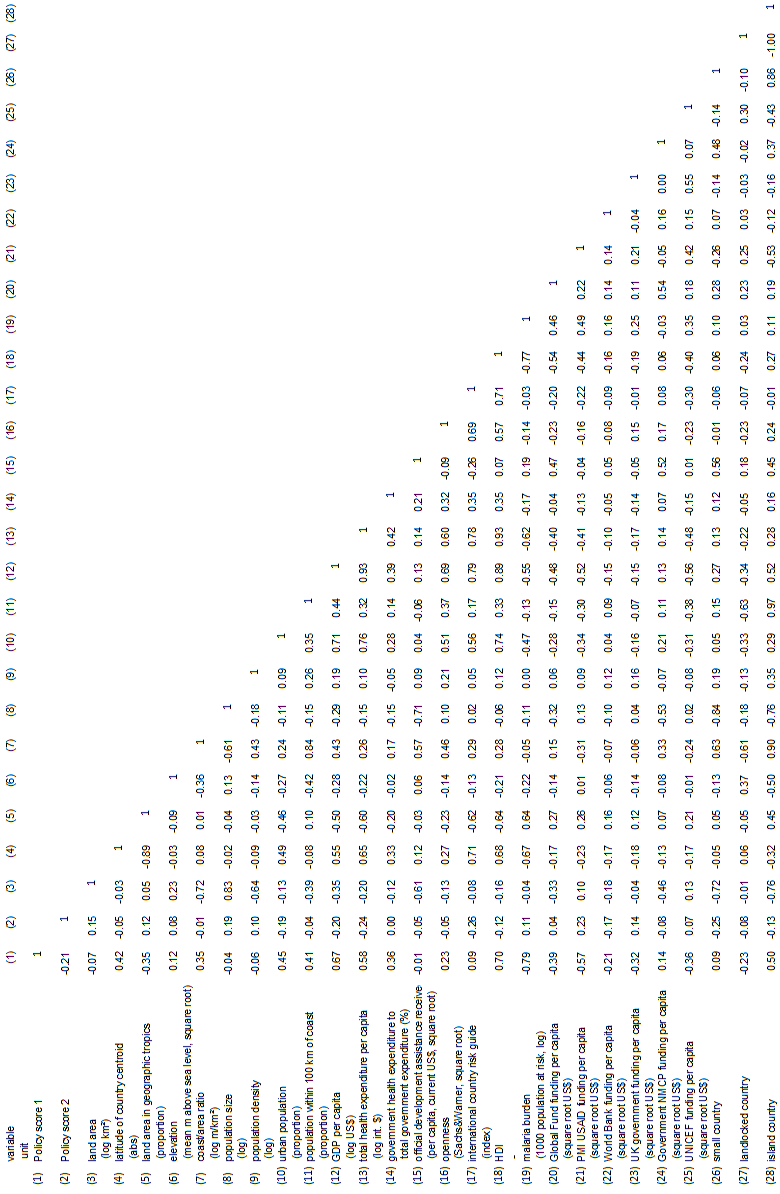


Additional file 4: Second policy score

| Country | Policy score 2 |
| --- | --- |
| Afghanistan | 0.910 |
| Algeria | 0.544 |
| Angola | 0.317 |
| Argentina | 0.544 |
| Bangladesh | 1.124 |
| Belize | 0.497 |
| Benin | 1.234 |
| Bhutan | 0.497 |
| Bolivia | 0.855 |
| Botswana | 0.497 |
| Brazil | 0.574 |
| Burkina Faso | 1.061 |
| Burundi | -0.108 |
| Cambodia | 0.567 |
| Cameroon | 0.789 |
| Cape Verde | 0.958 |
| Central African Republic | 0.057 |
| Chad | 0.885 |
| China | 0.343 |
| Colombia | 1.124 |
| Comoros | 1.192 |
| Congo | 0.723 |
| Costa Rica | 0.813 |
| Cote d'Ivoire | 0.231 |
| Democratic Republic of the Congo | 0.781 |
| Djibouti | 0.058 |
| Dominican Republic | 0.524 |
| Ecuador | 0.670 |
| El Salvador | 1.094 |
| Equatorial Guinea | 0.058 |
| Eritrea | 1.308 |
| Ethiopia | 1.365 |
| French Guiana | 0.723 |
| Gabon | 0.057 |
| Gambia | 0.885 |
| Ghana | 0.719 |
| Guatemala | 0.986 |
| Guinea | 1.058 |
| Guinea-Bissau | 0.336 |
| Guyana | 0.208 |
| Haiti | 0.264 |
| Honduras | 0.497 |
| India | 1.124 |
| Indonesia | 1.124 |
| Iran | 1.267 |
| Kenya | 0.723 |
| Laos | 0.567 |
| Liberia | 1.234 |
| Madagascar | 0.781 |
| Malawi | 0.723 |
| Malaysia | 0.764 |
| Mali | 0.509 |
| Mauritania | 0.490 |
| Mayotte | 0.310 |
| Mexico | 0.813 |
| Mozambique | 1.234 |
| Myanmar | 1.124 |
| Namibia | 1.267 |
| Nepal | 0.779 |
| Nicaragua | 0.986 |
| Niger | 0.142 |
| Nigeria | 0.547 |
| North Korea | 0.682 |
| Pakistan | 0.284 |
| Panama | 0.544 |
| Papua New Guinea | 0.820 |
| Paraguay | 0.229 |
| Peru | 1.439 |
| Philippines | 0.937 |
| Rwanda | 0.346 |
| Sao Tome and Principe | 0.504 |
| Saudi Arabia | 0.738 |
| Senegal | 1.234 |
| Sierra Leone | 1.234 |
| Solomon Islands | 0.317 |
| Somalia | 1.192 |
| South Africa | 0.586 |
| South Korea | -0.472 |
| South Sudan | 1.061 |
| Sudan | 0.654 |
| Suriname | 0.285 |
| Swaziland | 0.816 |
| Tanzania | 0.454 |
| Thailand | 0.772 |
| Timor | 1.095 |
| Togo | 0.719 |
| Uganda | 1.234 |
| Vanuatu | 0.779 |
| Venezuela | 0.497 |
| Vietnam | 0.986 |
| Yemen | 0.910 |
| Zambia | 1.234 |
| Zimbabwe | 1.391 |
