# Supplementary material for: A cross-country comparison of malaria policy as a premise for contextualized appropriation of foreign aid in global health
Source: Health Res Policy Syst. 2021 Jun 14;19:93. doi: 10.1186/s12961-021-00700-6 (PMC8201720; doi:10.1186/s12961-021-00700-6)
Supplement: Supplementary file 4 — Additional file 4. Second policy score. [file 12961_2021_700_MOESM4_ESM.pdf]

Health Research Policy and Systems

A Cross-Country Comparison of Malaria Policy as a Premise for Contextualized Appropriation of Foreign Aid in Global Health

--Manuscript Draft--

**Manuscript Number:**	HRPS-D-20-00344R1
**Full Title:**	A Cross-Country Comparison of Malaria Policy as a Premise for Contextualized Appropriation of Foreign Aid in Global Health
**Article Type:**	Research
**Funding Information:**	
**Abstract:**	Background: Foreign aid continues to play an essential role in health sector development of low-resource countries, especially constituting a vital portion of their health expenditures. However, the relationship between the foreign aid allocation and malaria policy formulation and/or implementation among state aid recipients remains unknown.<br>Methods: Publicly available data were collected with the country as observational unit to set up the conceptual framework. The quality and strength of relationships between socio-economic, environmental and institutional parameters were estimated by Pearson and polychoric correlations. A correlation matrix was explored by factor analysis.<br>Results: The first policy index captured policy variation related to malaria burden and development assistance. Funding per capita from all international agencies was correlated with malaria burden, whereas governmental funding for national malaria programmes per capita was not. The second policy index captured variation beyond malaria endemicity and country size. It was found to be related to international country risk instrument and funding from USAID President's Malaria Initiative.<br>Conclusions: Not all agencies involved in malaria policy allocate assistance in alignment with GDP and malaria burden. While the country size does not negatively impact malaria burden, it does account for greater development assistance per capita from selected international agencies. Novel policy indexes describe complex relationships between malaria policy, international foreign aid and socio-economic parameters. Small countries have distinct environmental and socio-political properties.
**Corresponding Author:**	Tomas Jezek<br>Masarykova Univerzita<br>CZECH REPUBLIC
**Corresponding Author E-Mail:**	tomas.jezek@volny.cz
**Corresponding Author Secondary Information:**	
**Corresponding Author's Institution:**	Masarykova Univerzita
**Corresponding Author's Secondary Institution:**	
**First Author:**	Tomas Jezek
**First Author Secondary Information:**	
**Order of Authors:**	Tomas Jezek
	Oluwaseun Adebayo Bamodu
**Order of Authors Secondary Information:**	
**Response to Reviewers:**	Dear Editors-in Chief,<br>We are grateful for the reviewers' feedback, and for the opportunity given us to revise our work. Making use of the reviewers' comments and critiques, we have revised our manuscript and provided a point-by-point response to the concerns raised by the reviewers. We believe these comments have helped improve the quality and appeal of our work. We hope the revised manuscript would be accepted for publication in the journal.

Powered by Editorial Manager® and ProduXion Manager® from Aries Systems Corporation

Sincerely,
Tomas Jezek, MD

Response to Reviewers:
Point-by-point responses to reviewer’s comments - Reviewer 1:
We thank the reviewer for carefully reading our manuscript and providing valuable comments. We believe making use of all these comments has further helped improve the quality and appeal of our work, as well as strengthened the manuscript. Below are our point-by-point responses.

Q1.1."Starting from the research question "The purpose of this study was to identify and understand key parameters that affect decision making and health policy implementation". The key word in the research question is the "understanding" and not statistical description in this senses there are several questions relating to the used methodology.

A1.1. We thank the reviewer for this comment. We have revised our manuscript extensively. To address the reviewer’s concern and for avoidance of doubts, we have omitted the alluded statement, especially as it gives room for misconstruing the objective of the present work, which is to make a case for case for proper nation-contextualization appropriation of foreign aids and to provide novel data-driven, statistics-based policy indexes that demystifies the interplay between the allocation of malaria control funds and implementation of national malaria intervention policies.

Q1.2. The parameters that could affect a decision-making process and health policy implementation aren't all the time quantitative and most of the time are qualitative. For example, two parameters can determine a policy decision: the availability of data and information to create a certain certainty to take the decision and the agreement among those involved in the decision to take. In this sense the methodology shouldn't concentrate only on the quantitative area but most of the analysis could have been done based on qualitative analyses. The qualitative analyses will provide precisions as to how decisions are taken (for the understanding dimension of the research question) and will give a meaning to the quantitative linkages and correlations. A document review could also have been performed to analyse documented analyses of stakeholders opinions.

A1.2.We sincerely thank the reviewer for these insightful comments. We do agree with the reviewer that (i) the availability of data and information to create a certain certainty to take the decision and (ii) the agreement among those involved in the decision to take are principal parameters that determine a policy decision; howbeit, the present study focused on the former, and was intended to be wholly quantitative. This is so because report of our second study which is in preparation is based on qualitative analyses of documented stakeholders opinions in foreign aid appropriation and malaria policy implementation. Please kindly see our revised manuscript.

Q1.3. If the research question was mainly focused on the quantitative side that represents only one side of the decision making set of determinants and precise the limitations that entails, that will make a sense.

A1.3. We thank the reviewer for this suggestion. As alluded in A1.2, we do agree with the reviewer that both quantitative and qualitative parameters determine a policy decision and implementation of same. The present study is by design intended to focus strictly on quantitative associations and correlations to provide a data-driven basis for proffering novel policy indexes that encapsulate the critical but complex relationships between foreign aids and the socio-economic parameters of recipient countries. Another from a parallel study which is wholly qualitative and preparationbased on qualitative analyses of documented stakeholders opinions in foreign aid appropriation and malaria policy implementation thus, our retention of the quantitative nature of the present manuscript. Please kindly see our revised manuscript.

Q1.4. In my opinion, this quantitative analysis won't give a clear answer on how these variables contributed to shape the decision making. It's like if we want to do a policy analysis or try to explain something that is by nature a implicit knowledge throughout

	quantitative parameters. Without a qualitative analysis that will complement, the research question will remain unanswered. There is a strong possibility that decisions were based on other parameters that have nothing to do with the analysed variables."<br><br>A1.4. We sincerely thank the reviewer for this comment. In our revised manuscript we have carefully and extensively revised for likely English language typographical and grammatical errors. We hope the reviewer finds our revised version more acceptable, keeping in mind that we have another study on same theme which is wholly qualitative in search for funding. Once again, we thank the reviewer.<br><br>Point-by-point responses to reviewer's comments - Reviewer 2:<br>We thank the reviewer for carefully reading our manuscript and providing valuable comments. We believe making use of all these comments has further helped improve the quality and appeal of our work, as well as strengthened the manuscript. Below are our point-by-point responses.<br><br>Q2.1.Thank you for performing this research on malaria funding. The article is insightful and provides useful evidence to policymakers and donors, especially that funding is not always aligned with malaria burden. The analysis appears to be carefully performed with the product being a well written article. The only parameter that I wished would have been included is country working language. (I have heard remarks that English-speaking countries are better at securing global health funding, and it would have been interesting to see if your data confirmed or refuted this.)<br><br>A2.1. We sincerely thank the reviewer for the kind appreciation of our work, and the encouraging comments provided. To address the reviewer's concern, we have now included data regarding aid appropriation and the English speaking status of recipient country in our revised manuscript. Please kindly see our revised manuscript, Page 9:<br><br>Analysis of international funding<br>Funding per capita from the Global Fund (r=0.45, p<0.01), USAID President's Malaria Initiative (PMI USAID) (r=0.49, p<0.01), United Kingdom of Great Britain and Northern Ireland (UK) government (r=0.25, p<0.05), and UNICEF (r=0.35, p<0.01) was positively correlated with malaria burden (Additional file 3). Funding per capita was found to be negatively correlated with the GDP (Global Fund: r=-0.50, p<0.01; UNICEF: r=-0.57, p<0.01; PMI USAID: r=-0.49, p<0.01) (Additional file 3).<br>The Global Fund funding per capita exhibited statistically significant but weak negative correlation with land area (r=-0.33; p<0.01) and population size (r=-0.37; p<0.01) (Additional file 3). Though statistically significant, Government NMP funding per capita showed moderately negative correlation with the land area (r=-0.46; p<0.01), and population size (r=-0.53; p<0.01), with Sao Tome and Principe being an outlier. The PMI USAID funding per capita was also found to exhibit significant negative correlation with latitude (r=-0.24; p<0.05). Moreover, consistent with speculations that the official language of recipient countries plays a role in the source and ease of securing global health funding, we found that there was significantly strong positive correlation between PMI USAID (r=0.26, p<0.05), UK funding (r=0.33, p<0.01) and English-speaking status of recipient countries.
**Additional Information:**	
**Question**	**Response**
Are you submitting this manuscript to a Thematic Series?	No

Powered by Editorial Manager® and ProduXion Manager® from Aries Systems Corporation

Manuscript

Click here to access/download;Manuscript;manuscript hrps.doc

Click here to view linked References

A Cross-Country Comparison of Malaria Policy as a Premise for Contextualized Appropriation of Foreign Aid in Global Health

Tomas Jezek, MD.[1*], Oluwaseun Adebayo Bamodu, MD., MS., PhD[2,3,4]

[1]Department of Preventive Medicine, Faculty of Medicine, Masaryk University, Brno 549 491 111, Czech Republic. tomas.jezek@volny.cz

[2]Department of Medical Research & Education, Taipei Medical University – Shuang Ho Hospital, New Taipei City 235, Taiwan. 16625@s.tmu.edu.tw

[3]Department of Hematology and Oncology, Taipei Medical University – Shuang Ho Hospital, New Taipei City 235, Taiwan

[4]Department of Urology, Taipei Medical University – Shuang Ho Hospital, New Taipei City 235, Taiwan

*Corresponding author

Abstract

Background: Foreign aid continues to play an essential role in health sector development of low-resource countries, especially constituting a vital portion of their health expenditures. However, the relationship between the foreign aid allocation and malaria policy formulation and/or implementation among state aid recipients remains unknown.

Methods: Publicly available data were collected with the country as observational unit to set up the conceptual framework. The quality and strength of relationships between socio-economic, environmental and institutional parameters were estimated by Pearson and polychoric correlations. A correlation matrix was explored by factor analysis.

Results: The first policy index captured policy variation related to malaria burden and development assistance. Funding per capita from all international agencies was correlated with malaria burden, whereas governmental funding for national malaria programmes per

capita was not. The second policy index captured variation beyond malaria endemicity and country size. It was found to be related to international country risk instrument and funding from USAID President's Malaria Initiative.

<u>Conclusions:</u> Not all agencies involved in malaria policy allocate assistance in alignment with GDP and malaria burden. While the country size does not negatively impact malaria burden, it does account for greater development assistance per capita from selected international agencies. Novel policy indexes describe complex relationships between malaria policy, international foreign aid and socio-economic parameters. Small countries have distinct environmental and socio-political properties.

Keywords: Environmental health, public health, global health, health care economics and organizations, health policy, international agencies, malaria

Background

Studies of economic development have found strong associations of geography and gross domestic product (GDP) per capita (1). Coastal economies are favourably located for foreign trade and have generally higher GDP than landlocked economies. Coastlines and areas connected to the coast by navigable waterways are more densely populated than the hinterlands. Most tropical countries are economically poor. Several studies suggest that economic development is fundamentally determined by the quality of institutions (2).

While the first foreign aid can be traced as far back as in to the 19th century, the flow of foreign aid globally has steadily increased after the World War II. The process of colonies attaining independence may have contributed to this change, however, a methodical shift from internal (within one administrative entity) to foreign transfer is considered a significant contributory factor too. Studies on the economic development of former colonies highlight the substantial impact of institutional development (2). Howbeit, some studies have suggested

that the historical colonial rule has insidiously evolved into indirect economic control exercised by international organizations, bilateral donors and (semi-)private investors, forming neo-colonial practices (3).

Many emerging or newly emerged states are strategically positioned and economically aligned, having joined international organizations regardless of their geographical or population size, thus amplifying the political, social and economic clout of such small countries beyond what the population size only would suggest. Analyses of foreign aid disbursement in the 1970s reveal that small countries received more foreign aid per capita than their larger counterparts. This so-called “small country effect” has been attributed to perceived and/or actual economic openness of small countries, which is probably linked to existent need for more aid to finance their imports (4). Other explanations see a major role of economic and political properties which small countries have taken from their history. Geographical area is not the lone parameter used in defining small states. According to the World Bank, countries with a population of 1.5 million or less, or membership in the Small States Forum are considered as small (5). Other definitions have been hinged on aggregated indicators (6), factor analysis (7) or cluster analysis (8).

In historical context, the relationship between socio-economic parameters and health contributed to the emergence of epidemiology as a standalone scientific discipline. This in itself has been the undertone in several studies depicting colonial history as a direct socio-political determinant of health (9), added to accrued evidence of similar impact by history of socialist systems, or conflicts and wars. The acclaimed elimination of malaria from wealthier countries in the first half of the 20th century was a result of both socio-economic development and intensive antimalarial interventions (10). However, up to the present time, malaria accounts for one of the highest disease burdens in global or public health, even when

prevention and treatment of malaria remain largely cost-effective public health interventions. The return on investments in malaria prevention and treatment could be as high as 40 times, compared with inaction (11), thus, the suggested strong association between a country's malaria burden and its economic development (1). This informs the need to include policies for malaria control in government poverty reduction agenda (12). Foreign aid for malaria control is largely provided by international organizations such as the Global Fund to Fight AIDS, Tuberculosis and Malaria (Global Fund), the World Bank or the United Nations Children's Fund (UNICEF). The US Agency for International Development (USAID) monitors the stability, recovery and democratic reforms in state recipients of foreign aid using the international country risk guide (ICRG) for political, economic and financial risk rating (13). Good understanding and informed re-evaluation of current allocation policy are critical for improving the effectiveness of foreign aid.

In most cases, health policy analyses performed ‘out-of-state’ tend to omit or ignore the political implications/ramifications of the public health agenda of interest. Since many of the countries affected by malaria have a colonial history, malaria is also subject to developmental policies. Policies are usually shaped through complex interactions of key stakeholders. However, the relationships between the environment, institutions, malaria as a disease entity, and health policy have rarely been explored. Little is known about the decision-making process for selecting and altering national malaria intervention policies. Given that national health policy on malaria is defined at the country level, we posit that cross-country comparisons could provide some needed insight into such health policy processes. While major decisive factors are often difficult to identify, it is highly probable that they vary between countries. This cannot be ascertained without robust analytical frameworks, which are currently lacking (14). Thus, necessitating the present study, which intends to identify and

demystify key parameters that affect decision making and health policy implementation, with particular reference to malaria policies. Results reported herein are intended to assist decision makers in the informed implementation and critical evaluation of public health interventions for malaria control.

Methods

Study model and working hypotheses

This study was based on a statistical analysis of the hypothesis, that environmental, socio-economic and institutional parameters affect a country's malaria intervention policy. Parameters reported at the country level that could be related to the malaria policy were identified from the literature review. The term ‘country’ herein is as defined by the International Organization for Standardization (15).

To aid hypotheses validation, a conceptual framework with parameters clustered into socio-economic, environmental and institutional groups was constructed (**Figure 1**). Socio-economic parameters included the GDP, national budget, economic stability, and the level of urbanization. Environmental parameters are related to geographic conditions such as temperature, latitude, elevation above sea level, and coastal and island locations. Institutional parameters include government policies, trade barriers, health services and policies, educational programmes, agricultural incentives and infrastructure projects (16).

National malaria intervention policy and strategy adoption vary between countries. This variability is related to characteristics that are not known or not directly observable. Factor analysis allows us to identify underlying relationships as it creates new factors with detectable relationships to latent parameters. Based on these factors we are able to distinguish countries based on health policies they have adopted.

Variables

Explanatory variables were derived from the study hypothesis and taken at the country level. Where per capita data was not available, corresponding values were generated using the total population of the country. The latitude component of the country centroid was measured in absolute terms. Total health spending refers to average expenditures on health per person, expressed in international dollar using purchasing power parity (PPP). Most variables were taken for the last available year 2016. Data for the ICRG index was extracted from previously published reports (1) in order to facilitate comparisons with our results.

Since the definition of a small country is not standardized, the status of small country was ascribed only if such country fulfilled all of the World Bank criteria with or without membership in the Small States Forum. Included countries are listed in the **Additional file 1**. The binary variable was set as 1 for countries fulfilling the 'small country' criteria, and as 0 for countries. The binary variable for being landlocked was set as 1 for countries with no access to the sea, and 0 for the other countries.

Openness, which is a measure of a country's macroeconomic policies that interfere with foreign trade, was measured by the proportion of years from 1965 to 1990 that a country was open to trade (17).

The international country risk guide (ICRG) is a widely used indicator of institutional quality. The ICRG index consists of five indicators adjudged highly relevant to the security of private property and the enforceability of contracts, namely, the frequency of contract repudiation, the risk of expropriation, corruption in the government, a tradition of law and order, and bureaucratic quality (18). Countries are scored 0-10 according to perceived institutional quality; with higher score indicating lower the risk.

The concept of human development index (HDI) was invented to measure social development

(19). The index is based on the average of three indicators, namely, life expectancy at birth, years of schooling for adults and expected years of schooling for children, as well as (log) GDP per capita.

The population at risk of malaria transmission was defined as the population living in areas where malaria transmission is reportedly endemic. The malaria incidence rate was defined as the number of malaria cases per 1000 people in the risk population per year. The rate of malaria incidence reflects the burden of malaria. The binary variable for malaria transmission was defined based on the malaria incidence 0 (absent) for countries with null incidence and 1 (present) for countries with an incidence greater than zero. The malaria national intervention policy and strategy adoption were transformed to dichotomous variables. The binary variable for health policy was set as 1 (policy present) or 0 (policy absent). In the absence of general recommendations for the use of intermittent preventive treatment in pregnancy (IPTp) outside of Africa (20), values for non-African countries were imputed as 0 (absent). Missing values were imputed as 0 (absent).

Data collection

We tested our hypotheses using dataset in which countries were the observational units. Parameters at the country level were assembled into a database. The data were collected from public source, including the World Health Organization (WHO), the World Bank, and the Center for International Development (**Additional file 2**). The data quality control of the primary sources was assumed sufficient to prevent missing data or selection bias.

Data analysis

Potential outliers were identified. Quantitative variables with skewed distributions were transformed using the common logarithm ($\log_{10}$) or the square root, as statistically

appropriate. Variables ≤ 0 were rescaled prior to transformation to avoid data loss.

The strength of the relationships between continuous variables was estimated by the Pearson correlation coefficient (*r*), and magnitudes classed as strong, moderate and weak based on *r* thresholds of 0.70, 0.50 and 0.20 in absolute terms, respectively.

The strength of the relationships between categorical variables was estimated by the polychoric correlation coefficient. The tetrachoric variant of the polychoric correlation was used to estimate the correlation between two dichotomous variables. Tetrachoric correlation uses the 2×2 contingency table as a double dichotomization of a bivariate standard normal distribution (21). Tetrachoric correlation coefficients were added to the correlation matrix and explored by factor analysis. The eigenvalue (λ) of factors extracted for further analysis was set as 1 (22). The resulting factors 1-6 constituted new policy scores 1-6.

Continuous and binary variables were compared using two sample t-tests. Statistical significance was determined using a two-sided significance level of α=0.05. Predictors of malaria outcome were analysed by the multiple linear regression with the following model:

$$Y \text{ (malaria outcome)} = \beta_0 + \beta_1 X_1 + \beta_2 X_2 + \gamma(X_1 * X_2) + \varepsilon$$

A similar probit model was used for the dependent binary variable of malaria transmission. Models were extended to include interaction terms and possible confounding factors to avoid multicollinearity. The goodness of model fit was measured by the R-squared or pseudo R-squared statistic as appropriate.

Consistency checks and statistical analyses were conducted using Stata 14 software (StataCorp. 2015. Stat Statistical Software: Release 14. College Station, TX: StataCorp LP).

Results

Descriptive statistics

The majority of variables were found to have skewed distribution and were transformed by either taking the logarithm or square root. The basic variable characteristics are described in terms of means and standard deviations for continuous variables (**Table 1**) or counts and proportions for categorical variables (**Table 2**).

Analysis of international funding

Funding per capita from the Global Fund (r=0.45, $p<0.01$), USAID President's Malaria Initiative (PMI USAID) (r=0.49, $p<0.01$), United Kingdom of Great Britain and Northern Ireland (UK) government (r=0.25, $p<0.05$), and UNICEF (r=0.35, $p<0.01$) was positively correlated with malaria burden. Funding per capita was found to be negatively correlated with the GDP (Global Fund: r=-0.50, $p<0.01$; UNICEF: r=-0.57, $p<0.01$; PMI USAID: r=-0.49, $p<0.01$) (**Additional file 3**).

The Global Fund funding per capita exhibited statistically significant but weak negative correlation with land area (r=-0.33; $p<0.01$) and population size (r=-0.37; $p<0.01$) (**Additional file 3**). Though statistically significant, Government NMP funding per capita showed moderately negative correlation with the land area (r=-0.46; $p<0.01$), and population size (r=-0.53; $p<0.01$), with Sao Tome and Principe being an outlier. The PMI USAID funding per capita was also found to exhibit significant negative correlation with latitude (r=-0.24; $p<0.05$). Moreover, consistent with speculations that the official language of recipient countries plays a role in the source and ease of securing global health funding, we found that there was significantly strong positive correlation between PMI USAID (r=0.26, $p<0.05$), UK funding (r=0.33, $p<0.01$) and English-speaking status of recipient countries.

Analysis of country size

Small countries did not seem to differ significantly from large countries in their malaria burden [diff = 0.28 (-2.86; 3.42); p=0.85] (data not shown). Being a small country showed strong positive correlation with being an island, moderate positive correlation with official development assistance received per capita, including Global Fund and government NMP funding for malaria control per capita, and weak positive correlation with population density. Conversely, being a small country exhibited strong negative correlation with population size and land area, but weak negative correlation with PMI USAID funding per capita (**Additional file 3**).

Malaria intervention policies and strategy adoption

Binary indicators for malaria national intervention policies and strategy adoption (**Table 5**) were used for the factor analysis. From the analysis, factors 1 to 6 which had eigenvalues > 1, accounted for 89% of the total variation. Factor 1 and factor 2 were not found to be clustered and their highest loadings exceeded the statistically strong absolute value of 0.70. Factors 1-4 had three or more variables with loadings above (absolute) 0.50. The relationships between the factors, malaria burden, and the social, economic and environmental variables are listed in Additional file 3.

Policy scores

As shown in **Additional file 3**, Policy score 1 showed moderate positive correlation with the HDI (r=0.69; p<0.01), GDP per capita (r=0.66; p<0.01), and total health expenditure per capita (r=0.66; p<0.01), mild positive correlation with being an island (r=0.49; p<0.01), urban population share (r=0.46; p<0.01), the latitude (r=0.41; p<0.01), population within 100 kilometres of the coast (r=0.38; p<0.01), the proportion of government health expenditure to

total government expenditure (r=0.36; $p<0.01$) and economic openness (r=0.23; $p<0.05$). In contrast, though statistically significant, Policy score 1 exhibited strong negative correlation with the malaria burden (r=-0.78; $p<0.01$), moderate negative correlation with PMI USAID funding (r=-0.57; $p<0.01$), and weak negative correlation with Global Fund aid (r=-0.38; $p<0.01$), World Bank aid (r=-0.20; $p<0.05$), UK government funding (r=-0.31; $p<0.01$), UNICEF funding (r=-0.36; $p<0.01$) per capita and being a landlocked country (r=-0.23; $p<0.05$). Howbeit, Policy score 1 was not significantly related to government NMP funding per capita or being a small country.

As depicted in **Figure 3**, Policy score 2 showed weakly negative correlation with the ICRG index (r=-0.26; $p<0.05$), PMI USAID funding per capita (r=0.21; $p<0.05$) and being a small country (r=-0.27; $p<0.01$). Policy score 2 was not significantly correlated with GDP per capita or the malaria burden.

Policy score 3 exhibited weak negative correlation with the proportion of *Plasmodium vivax* (r=-0.27; $p<0.01$) and elevation above sea level (r=-0.24; $p<0.05$). Since the prevalence of *Plasmodium falciparum* outside Africa is generally below 5%, policy score 3 could also be considered as representing malaria policy outside Africa. Policy score 4 was statistically significant and weakly correlated with openness (r=0.28; $p<0.05$) and PMI USAID funding per capita (r=0.21; p<0.05).

Discussion

The primary objective of the study was to identify key parameter(s) that influence the allocation of funding for malaria control and implementation of national malaria intervention policies. The novel policy indexes presented herein reflect not only malaria endemicity, but also ‘epi-endemicity’ parameters.

International funding

Hitherto, the commitment of international agencies has determined the success of malaria control programmes, with such international aid accounting for approximately 68% of global funding for malaria. The largest share of malaria funding (40% of total funding) is provided by The Global Fund, followed by the USAID President's Malaria Initiative (PMI) (26%), the UK government (7%) and the World Bank (3%). Both the UK government and the PMI USAID also contribute to the Global Fund. In the last decade, multinational organizations have steadily increased their global health agenda support, such that their contribution is purportedly higher than those of organizations that were specifically established to promote global health agenda such as the WHO.

The presented outcomes suggest that funding for malaria control per capita is inconsistently distributed depending on the funding provider. The populace o smaller countries and countries with smaller populations benefit disproportionally from malaria control funding. Only PMI USAID funding is related to the institutional quality of the recipient country.

Policy indexes

First policy index describes the endemicity of malaria. Second policy index indicates economic and institutional conditions in 93 countries and represents the variation in national malaria intervention policy that is not nor can be explained by malaria endemicity (Additional file 4). Since the second policy score is related to the size of the country but not to malaria or GDP, this score explains novel components of the small country bias. The quality of institutions is adversely affected by corruption, thus reducing corruption improves the institutional quality and often translates into more efficient healthcare systems (23). Corruption is negatively correlated with public spending on education and health, and even

with total health expenditure per capita (24). Improving the quality of national institutions seems to increase the average personal spending on health (25).

Small countries

High costs of public service, telecommunication and transportation impede the delivery of health care, education and infrastructure services in small countries. Many governments are implementing health system reforms in order to improve health services and health financing. International agencies support these reforms through various development policies. Our results do not support the claim that small countries are disadvantaged in the allocation of GDP per capita or that they face a higher malaria burden. Nevertheless, small countries receive more foreign aid or international development assistance per capita. This may be attributed, in part, to the high cost of delivering this development assistance due to poor geographic conditions. It is also possible that different allocation criteria are employed for these small countries. Interestingly, although, many small countries experience the emergence and re-emergence of vector-borne diseases (26), according to our data, malaria is more likely to increase in larger countries than in their smaller counterparts.

Limitations

The strength of any data analysis depends on the accuracy of the data. The specific ecosystem of malaria research utilizes data from many sources of varying quality. Nevertheless, we have used the same data sources as used by the WHO, so we assume that the data quality is reasonable enough to address our questions. Most variables were derived from public datasets from 2016; however some data generated by private organisations had restricted access, being freely available only for selected past years.

Conclusions

The present study proffers novel policy indexes that encapsulate the critical but complex relationships between foreign aid and the socio-economic parameters of recipient countries. The second policy index is independent of malaria endemicity and provides novel insight into malaria policy formulation and/or implementation.

Our findings support the indispensability of fiscal transparency and data-driven approach, as well as reveal that not all involved agencies allocate funding for malaria control based on GDP or malaria burden. These results provide a basis for and encourage further studies of the interplay between the allocation of malaria control funds and implementation of national malaria intervention policies. Having these results from the country level could contribute to better allocation of malaria funding.

The small country effect is deeply entrenched in international developmental policies following claims of state economic disparity and small country disadvantages. However, our study did not confirm disadvantages in the social and economic resources of small countries if parameters are analysed per capita. The country size bias does account for greater development assistance per capita from selected international agencies. Awareness of all these highlight the need for better adjustment of aid allocation to the distinct needs of small countries.

List of abbreviations

ACT: artemisinin-based combination therapy;

DDT: dichloro-diphenyl-trichloroethane;

G6PD: glucose-6-phosphate dehydrogenase;

GDP: gross domestic product;

Global Fund: Global Fund to Fight AIDS, Tuberculosis and Malaria;

HDI: human development index;

ICRG: the international country risk guide;

IM: intramuscular;

IPTc: intermittent preventive treatment in children;

IPTp: intermittent preventive treatment in pregnancy;

IRS: indoor residual spraying;

ITN: insecticide-treated mosquito net;

LLIN: long-lasting insecticidal net;

log: common logarithm;

NMP: national malaria programme;

PPP: purchasing power parity;

PMI: the US President's Malaria Initiative;

RDT: rapid diagnostic test;

SMC: seasonal malaria chemoprevention;

UK: United Kingdom of Great Britain and Northern Ireland;

USAID: US Agency for International Development;

UNICEF: United Nations Children's Fund;

WHO: World Health Organization

Declarations

Ethics approval and consent to participate: Not applicable.

Consent for publication: Not applicable.

Availability of data and material: The datasets used and/or analysed during the current study are available from the corresponding author on reasonable request.

Competing interests: Authors declare that they have no competing interests.

Funding: Not applicable.

Authors' contributions: TJ: Study conception and design, collection and assembly of data. TJ, OAB: Data analysis and interpretation, Figure design, Manuscript writing. Both authors read and approved the final manuscript.

Acknowledgements: The authors are grateful to the reviewers of this paper for their helpful comments.

References

1. Gallup JL, Sachs JD, Mellinger AD. Geography and economic development. Int Reg Sci Rev. 1999;22(2):179–232.

2. Easterly W, Levine R. Tropics, germs, and crops: how endowments influence economic development. J Monet Econ. 2003;50(1):3–39.

3. Braveman PA. Epidemiology and (neo-) colonialism. J Epidemiol Community Health. 2001;55(3):160–161.

4. Dudley L, Montmarquette C. A model of the supply of bilateral foreign aid. Am Econ Rev. 1976;66(1):132–142.

5. Crowards T. Defining the category of ‘small’states. J Int Dev. 2002;14(2):143–179.

6. Jalan B. Problems and Policies in Small Countries. Lond Croom Helm. 1982;

7. Downes AS. On the statistical measurement of smallness: a principal component measure of country size. Soc Econ Stud. 1988;75–96.

8. Schiavo-Campo S. Some considerations on development aid to small countries. Dev Policy Small States Selwyn P Ed Croom Helm Lond. 1975;

9. Prussing E. Critical epidemiology in action: research for and by indigenous peoples. SSM-Popul Health. 2018;6:98–106.

10. Sachs J, Malaney P. The economic and social burden of malaria. Nature. 2002;415(6872):680.

11. World Health Organization. Action and Investment to Defeat Malaria 2016–2030-for a Malaria-Free World. Technical report, Geneva; 2016.

12. Okorosobo T, Okorosobo F, Mwabu G, Orem JN, Kirigia JM. Economic burden of malaria in six countries of Africa. Eur J Bus Manag. 2011;3(6):42–63.

13. Tarnoff C. US Agency for International Development (USAID): Background, operations, and issues. Congressional Research Service Washington, DC; 2015.

14. Williams HA, Durrheim D, Shretta R. The process of changing national malaria treatment policy: lessons from country-level studies. Health Policy Plan. 2004;19(6):356–370.

15. ISO - International Organization for Standardization [Internet]. ISO. [cited 2020 Oct 15]. Available from: https://www.iso.org/home.html

16. Hagenlocher M, Castro MC. Mapping malaria risk and vulnerability in the United Republic of Tanzania: a spatial explicit model. Popul Health Metr. 2015;13(1):2.

17. Sachs JD, Warner A, \AAslund A, Fischer S. Economic reform and the process of global

integration. Brook Pap Econ Act. 1995;1995(1):1–118.

18. Knack S, Keefer P. Institutions and economic performance: cross-country tests using alternative institutional measures. Econ Polit. 1995;7(3):207–227.

19. Sagar AD, Najam A. The human development index: a critical review1. Ecol Econ. 1998;25(3):249–264.

20. Global Malaria Programme WHO. Policy Brief for the Implementation of Intermittent Preventive Treatment of Malaria in Pregnancy using Sulfadoxine-Pyrimethamine (IPTp-SP). 2013;

21. Ekström J. The phi-coefficient, the tetrachoric correlation coefficient, and the Pearson-Yule Debate. 2011;

22. Yeomans KA, Golder PA. The Guttman-Kaiser criterion as a predictor of the number of common factors. The Statistician. 1982;221–229.

23. Liang L-L, Mirelman AJ. Why do some countries spend more for health? An assessment of sociopolitical determinants and international aid for government health expenditures. Soc Sci Med. 2014;114:161–168.

24. Hay SI, Guerra CA, Tatem AJ, Noor AM, Snow RW. The global distribution and population at risk of malaria: past, present, and future. Lancet Infect Dis. 2004;4(6):327–336.

25. Gupta S, Davoodi H, Tiongson E. Corruption and the provision of health care and education services. In: The political economy of corruption. Routledge; 2001. p. 123–153.

26. Dave S, Dave P, Pal M. The impact of climate change on the emergence and re-emergence of vector borne human diseases. Int J Livest Res. 2015;5:1–10.

Tables

Table 1: Descriptive statistics of continuous variables

Variable *	Units	N	Mean	SD	Min	Max
Land area	log of km^2	215	4.72	1.31	0.30	7.22
Latitude of country centroid	absolute degree	164	27.35	17.78	0.42	74.70
Elevation above sea level	mean m	164	626.92	560.98	9.17	3185.92
Population size	log	215	6.59	1.06	4.05	9.13
Population density	log	195	1.9	0.62	0.29	4.41
Urban population	proportion	215	0.6	0.24	0.12	1.00
Population within 100 km of coast	proportion	164	0.43	0.36	0.00	1.00
GDP per capita PPP	log of US$	192	4.07	0.5	2.9	5.06
Total GDP PPP	log of millions US$	192	10.79	1.04	7.62	13.28
Total health expenditure per capita PPP	log of intl $	185	2.83	0.57	1.47	3.99
Government health to total government expenditure	proportion	186	3.56	2.34	0.37	13.06
Official development assistance received per capita	current US$	139	124.24	318.11	-2.34	3034.15
Openness (Sachs&Warner)	-	139	0.25	0.40	0.00	1.00
International country risk guide index	-	98	5.68	2.26	2.27	9.98
HDI	-	188	0.69	0.15	0.35	0.94
Malaria incidence	1000 population at risk	105	96.03	129.49	0.05	460.90
Plasmodium vivax species	proportion	105	0.27	0.37	0.00	1.00
Global Fund funding for malaria control per capita	US$	90	0.88	1.82	-0.07	14.85
PMI USAID funding for malaria control per capita	US$	90	0.27	0.55	0.00	3.10
World Bank funding for malaria control per capita	US$	91	0.01	0.03	-0.01	0.20
UK development funding for malaria control per capita	US$	90	0.04	0.20	0.00	1.29
Government funding for malaria control per capita	US$	70	0.80	2.98	0.00	24.89
UNICEF funding for malaria control per capita	US$	61	0.03	0.13	0.00	1.04
Policy score 1	-	93	0.08	0.40	-0.69	0.82
Policy score 2	-	93	0.73	0.39	-0.47	1.44
Policy score 3	-	93	-0.18	0.40	-0.87	0.92

Policy score 4	-	93	1.02	0.39	-0.05	1.84

* SD: standatd deviation; GDP: gross domestic product; HDI: human development index; Global Fund: Global Fund to Fight AIDS, Tuberculosis and Malaria; NMCP: national malaria control programme; PMI: United States President's Malaria Initiative; PPP: purchasing power parity; UK: United Kingdom of Great Britain and Northern Ireland government; UNICEF: United Nations Children's Fund; USAID: United States Agency for International Development; log: common logarithm; funding for malaria control (except UNICEF) as reported by donors.

Table 2: Descriptive statistics of categorical variables

**Variable ***	**N**	**Proportion of countries**
Small country dummy	215	0.35
Landlocked country dummy	149	0.23
Island dummy	96	0.64
English language dummy	215	0.42
ITNs/LLINs are distributed free of charge	93	0.89
ITNs/LLINs are distributed to all age groups	93	0.75
ITNs/LLINs are distributed through mass campaigns to all age groups	93	0.75
IRS is recommended by malaria control programme	93	0.90
DDT is used for IRS	93	0.10
IPTp is used to prevent malaria during pregnancy	93	0.40
Seasonal malaria chemoprevention (SMC or IPTc) is used	93	0.11
Patients of all ages should get diagnostic test	93	0.99
Malaria diagnosis is free of charge in the public sector	93	0.86
RDTs are used at community level	93	0.56
G6PD test is recommended before treatment with primaquine	93	0.19
ACT for treatment of *Plasmodium falciparum*	93	0.99
Pre-referral treatment with quinine or artemether IM or artesunate suppositories	93	0.59
Single dose of primaquine is used as gametocidal medicine for *P. falciparum*	93	0.47
Primaquine is used for radical treatment of *Plasmodium vivax* cases	93	0.57
Directly observed treatment with primaquine is undertaken	93	0.29

* ACT: artemisinin-based combination therapy; DDT: dichloro-diphenyl-trichloroethane; G6PD: glucose-6-phosphate dehydrogenase; IM: intramuscular; IPTc: intermittent preventive treatment in children; IPTp: intermittent preventive treatment in pregnancy; IRS: indoor residual spraying; ITN: insecticide-treated mosquito net; LLIN: long-lasting insecticidal net; RDT: rapid diagnostic test; SMC: seasonal malaria chemoprevention.

Table 3: Logistic regression model of any malaria transmission

independent variables (unit)	any malaria transmission (95% CI)
Land area	1.531
(log km^2)	(0.688 - 2.374)**
Latitude of country centroid	-0.151
(abs)	(-0.210 - -0.091)**
GDP per capita	-3.099
(log US$)	(-4.514 - -1.685)**
Pseudo R2	0.68
N	157

* p<0.05; ** p<0.01; CI: confidence interval; dependent variable: malaria transmission. A country is considered having malaria transmission if malaria incidence is greater than zero.

Table 4: Multiple linear regression model of malaria burden

independent variables	malaria burden (log)	malaria burden (log) with small country dummy
(unit)	(95% CI)	(95% CI)
Population density	-0.349	-0.351
(log)	(-0.669 - -0.030)*	(-0.669 - -0.007)*
Latitude of country centroid	-0.067	-0.067
(abs)	(-0.081 - -0.052)**	(-0.080 - -0.051)**
Elevation	-0.022	0.022
(mean m above sea level, square root)	(-0.040- -0.004)*	(-0.040- -0.004)*
GDP per capita	-1.498	-1.497
(log US$)	(-1.854 - -1.142)**	(-1.857 - -1.137)**
Small country	-	0.013
(dummy)		(-0.622 - 0.595)
R2	0.698	0.694
N	91	91

* *p*<0.05; ** p<0.01; CI: confidence interval; dependent variable: malaria burden.

Table 5: Malaria national intervention policy and factor loadings

malaria national intervention policy and strategy adoption	Factor1	Factor2	Factor3	Factor4	Factor5	Factor6
ITNs/LLINs are distributed free of charge	-0.31	-0.22	-0.59	0.56	0.32	0.06
ITNs/LLINs are distributed to all age groups	0.03	0.58	-0.11	0.66	0.01	-0.40
ITNs/LLINs distributed through mass campaigns to all age groups	-0.47	0.41	-0.5	0.18	0.29	-0.31
IRS is recommended by malaria control programme	-0.16	0.68	0.11	-0.47	0.19	-0.34
DDT is used for IRS	-0.03	0.57	0.47	-0.04	0.61	0.25
IPTp is used to prevent malaria during pregnancy	-0.66	0.05	0.6	0.38	-0.15	0.06
Seasonal malaria chemoprevention (SMC or IPTc)	-0.67	-0.08	0.65	0.26	-0.02	-0.02
Patients of all ages should get diagnostic test	0.55	0.01	0.66	0.5	-0.05	0.03
Malaria diagnosis is free of charge in the public sector	0.67	0.21	0.05	0.33	0.43	0.34
RDTs are used at community level	-0.41	0.35	-0.43	-0.11	-0.07	0.61
G6PD test is recommended before treatment with primaquine	0.40	0.25	-0.31	0.49	-0.54	0.11
ACT for treatment of Plasmodium falciparum	-0.40	0.82	0.08	-0.08	-0.37	0.02
Pre-referral treatment with quinine or artemether IM or artesunate supp	-0.70	0.32	-0.19	0.15	-0.12	0.21
Single dose of primaquine is used as gametocidal medicine for Plasmodium falciparum	0.68	0.53	-0.01	-0.05	0.02	0.1
Primaquine is used for radical treatment of Plasmodium vivax	0.91	0.01	-0.17	0.11	0.05	-0.13
Directly observed treatment with primaquine is undertaken	0.66	0.43	0.10	-0.22	-0.29	0.04

* ACT: artemisinin-based combination therapy; DDT: dichloro-diphenyl-trichloroethane; G6PD: glucose-6-phosphate dehydrogenase; IM: intramuscular; IPTc: intermittent preventive treatment in children; IPTp: intermittent preventive treatment in pregnancy; IRS: indoor residual spraying; ITN: insecticide-treated mosquito net; LLIN: long-lasting insecticidal net; RDT: rapid diagnostic test; SMC: seasonal malaria chemoprevention; factor loadings: factor 1-6

Figures

Figure 1: Conceptual framework of malaria burden

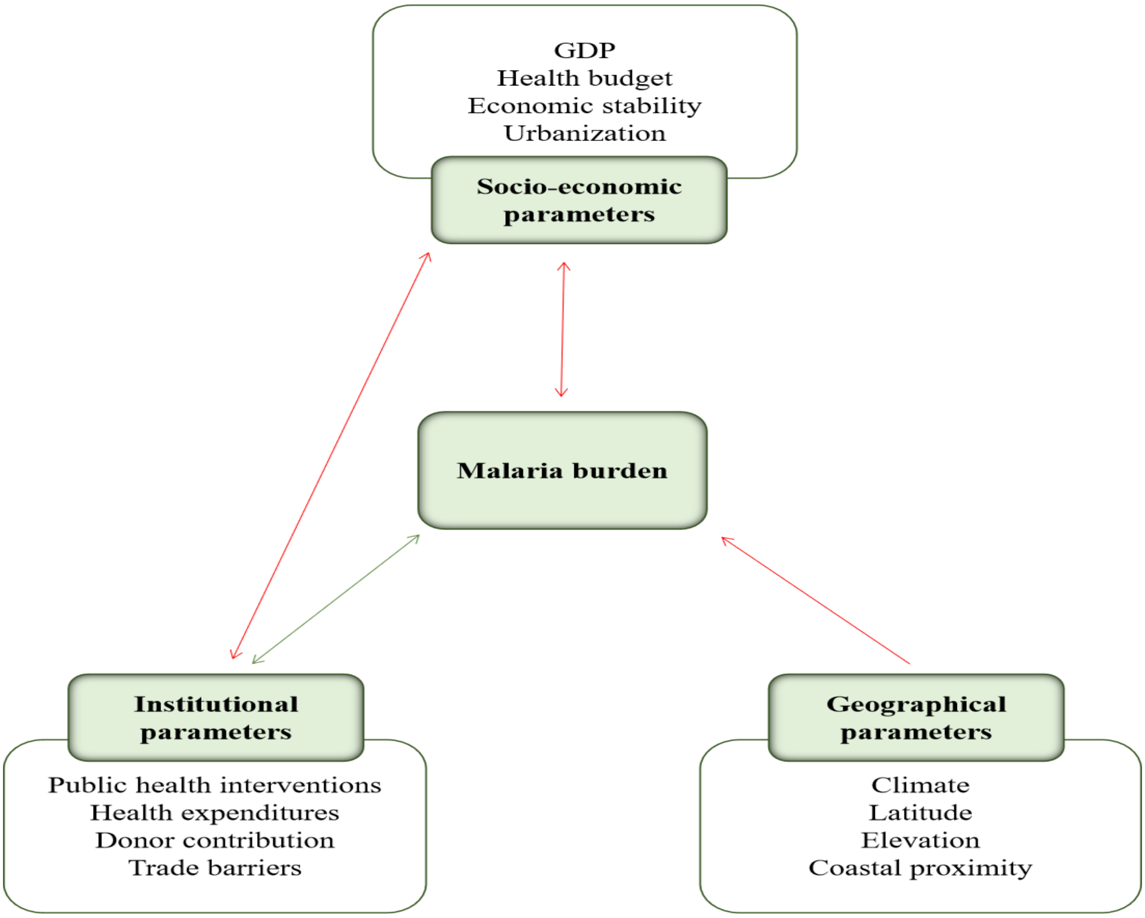

* framework categories (socio-economic, institutional and environmental) with corresponding parameters
a green line indicates a positive correlation, a red line indicates a negative correlation of parameters with malaria burden
GDP: gross domestic product; HDI: human development index; PMI: United States President's Malaria Initiative; UK: United Kingdom of Great Britain and Northern Ireland; UNICEF: United Nations Children's Fund; USAID: United States Agency for International Development;

Figure 2: Relationships of malaria burden and social, economic and environmental

parameters

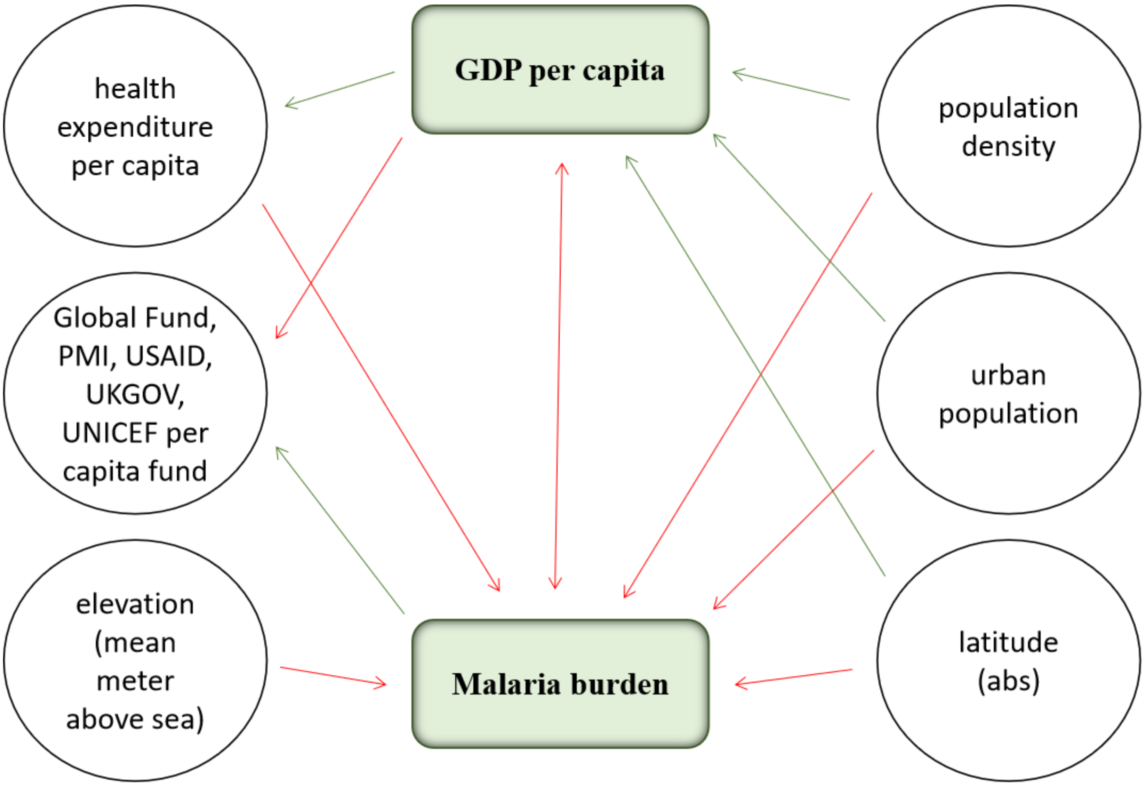

* green line indicates a positive correlation, red line indicates a negative correlation, yellow line indicates an interaction between parameters

GDP: gross domestic product; HDI: human development index; PMI: United States President's Malaria Initiative; UK: United Kingdom of Great Britain and Northern Ireland; UNICEF: United Nations Children's Fund; USAID: United States Agency for International Development;

Figure 3: Relationships of the second policy score and economic, institutional and environmental parameters

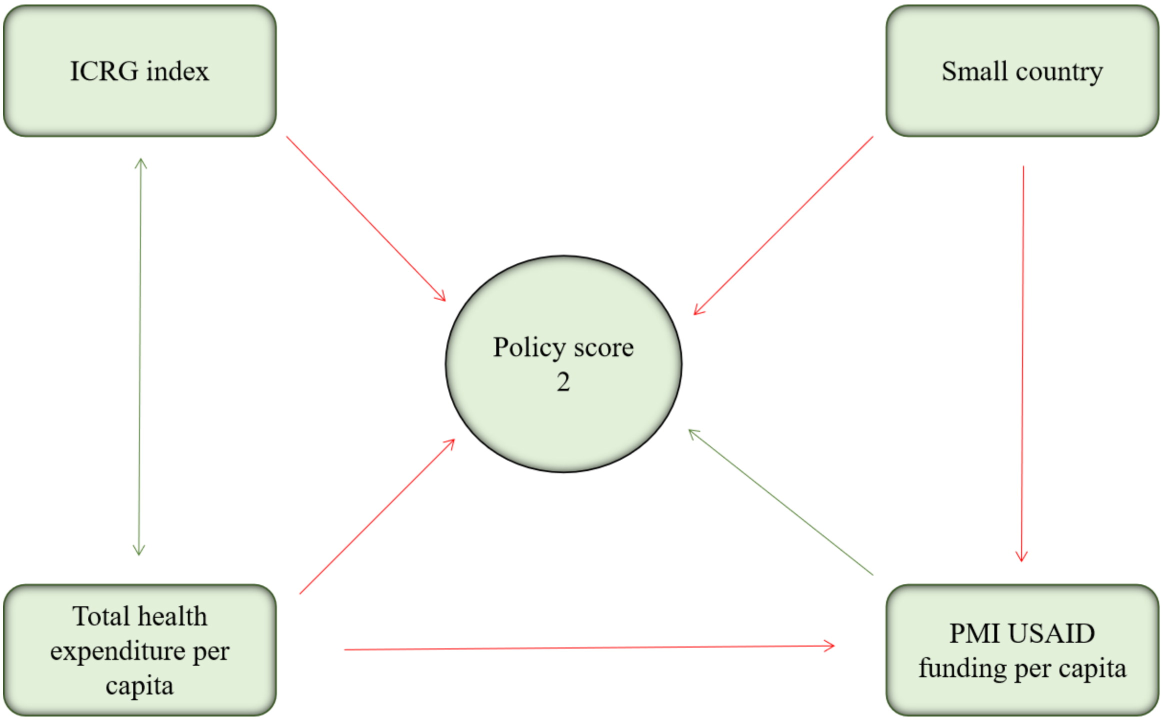

* green line indicates a positive correlation, red line indicates a negative correlation with policy score 2
ICRG: the international country risk guide; PMI: United States President's Malaria Initiative; USAID: United States Agency for International Development;

Supplementary Material

Additional file 1: Small countries and dependent territories

Additional file 2: Data sources

Additional file 3: Correlation matrix

Additional file 4: The second policy score

Click here to access/download
Supplementary Material
additional files hrps.doc
